# A Case of Hystero-Epilepsy

**Published:** 1896-09

**Authors:** Bonville B. Fox


### A CASE OF HYSTERO-EPILEPSY.

BY

BONVILLE B. FOX, M.A., M.D. Oxon.

---

THE claims of this case for report appear to me to rest: (1) on the unusual character, especially in the male sex, of its symptoms; (2) on the varying interpretation physicians have placed on those symptoms. These remarks refer exclusively to the physical phenomena. There was nothing in the mental symptoms calling for any special notice.

The subject was an unmarried man, aged 31, who had been farming in the Colonies for some time past.

*Family History*.—His father suffered from queer kind of seizures, in which he turned pale, lost consciousness, and uttered a cry, suggesting epilepsy; while there was a more vague notion that a maternal aunt had fits.

*Previous History*.—He has been steady and sober, free from syphilis, rheumatic fever, or chorea, and if not sharp at school, not conspicuously the reverse. In disposition he was perhaps rather uncertain, a little prone to be solitary and to sulk. There is an ill-defined history of an anomalous kind of fit twenty years ago. Till about September, 1891, he had enjoyed good health. Then, while working on a hay cart in America, he suddenly fell down, got up, but again fell down, this time unconscious. He was taken to a hospital, and when consciousness returned some paralysis was found. He remained in the hospital for more than six months, very much in the same state, was pointed out as a very interesting case, given any quantity of iodide of potassium, and finally discharged as incurable. During the voyage home it appears that he was able to move about so freely that his fellow-passengers hardly realised that he was an invalid, and this continued after he reached home. He could go up and down stairs, but was liable to collapse suddenly and completely, and at all times, however well he was moving, any sudden excitement would reduce him to helplessness. He had been liable to lose the thread of his conversation and to become confused for some time past.

On September 7th, 1892, when he was unusually bright and jolly, he suddenly burst out into violent laughing and sobbing, and soon became so violent and maniacal that he had to be tied down, and so remained until brought to Brislington House Asylum. On the night he arrived he was fed by the stomach tube; but his stomach rejected everything, and nourishment had to be administered by the rectum. The catheter was necessary. Examination of the urine and the organs generally returned negative results. Temperature normal. Forehead rather low and sloping. Palate arched. Pupils fixed, not reacting to light. He slept a great deal, and while sleeping there was considerable oscillation of both eyelids, but no other tremor. Immediately, however, on waking, tremor of the whole body began. When he attempted to take food or to answer questions strange attacks were induced. He lost self-control, rolled over and over, invariably towards the right side, and burst out into loud hysterical laughter, and at first vomited the food he had recently taken, while there was considerable jactitation both of body and limbs. This after a time passed away, and he at once went to sleep. The same thing occurred when he was touched, and especially when being washed, and he was so hyperæsthetic that this operation seemed to give him real pain. The whole body became quite rigid during the fit. He was unable to stand; his legs were somewhat emaciated, his feet extended—somewhat resembling the posture of equino-varus,—the patellar tendon-reflex was markedly exaggerated on both sides, but there was no ankle-clonus. There was slight paresis of the right arm, the power of grasping comparing unfavourably with that of its fellow. There was little power of speech, and he indicated that the back of his head was the seat of pain.

He improved under simple treatment, and in three weeks was free from the fits, save at meal times, when they always recurred, though he forgot all about their recurrence. During each of them the whole body became rigid, but the tongue was never bitten, the mouth being kept wide open; sickness sometimes occurred. As regards tremor, distinct nystagmus has been noted; but tremor generally was now absent as long as the patient was quiet, but began at once when he was spoken to, or when he thought he was being watched. He slept a good deal.

On October 6th, just a month after admission, he had a series of fits lasting two and a-half hours, followed by sleep. During the attacks the patient perspired freely; while his penis was in a state of erection. There was not the same rigidity of muscles noted before, and while the left arm was being continually flexed and extended, the right hand was picking at the bedclothes. After one or more fits he always appeared unconscious of anything untoward having happened, but he stated that always beforehand he suffered severe pain on the top of his head.

By the end of October, two months after admission, he had sufficiently regained power to be up and about, the tremor was less, the laughing and violent fits absent, but he complained of cutting pains in the right thigh. But on November 17th, after the usual prodromal headache, he had a series of seizures, and on the 18th a remarkable fit, in which the left arm and leg were thrown into a series of convulsions—at first tonic, then clonic—indistinguishable from those of epilepsy; the tongue was protruded to its fullest extent and was very tremulous. It remained in this position some minutes after consciousness returned, the patient intimating he had lost the power to retract it. There was great twitching of the supra-orbital muscles. Water and a motion had been passed shortly before the fit, but not during its continuance. Afterwards for quite an hour he suffered from marked dyspnœa, and his pulse was very rapid and small. He was unable to speak, but was not truly aphasic, as the idea of language was intact, and he could indicate his meaning by pointing to the letters. Nor was he strictly aphonic, if, as some authorities state, aphonic patients can whisper. He certainly could not utter a sound. This attack was followed by loss of power in the legs, which kept him upstairs a fortnight. During this time he occasionally had pain in the head associated with dyspnœa and with chokiness (globus), and once with shooting pain in the right eye. Then he did well until January 13th, 1893, when his brother paid him a visit. This was followed by a bad night—a series of laughing fits, pain in the head, inability to articulate or to walk. In a fortnight's time he still had no power in his legs; he could grip well with his hands, but could not speak. By February 9th, about a month from the attack, he was able to walk and to speak, the latter not quite well. It is now noted that the knee-reflex is natural on the left side, though exaggerated on the right. He went on pretty well for some weeks, though he complained of a queer feeling at the back of his tongue at meal times, which made him wish to cry out and to stop eating. He occasionally lost his speech after pain on the top of his head, and on several nights had to get up many times to pass water. On the 18th of March he drove into Bristol, and though on starting he was in fair health and able to walk to the carriage, he could not move a step on his return. He quickly regained power, and two days subsequently attended our chapel service, where he had a seizure just like a faint, save that the tongue was greatly protruded, followed by a series of fits with laughter and jactitations, followed in turn by prolonged sleep. The next day he could not speak, but indicated that he had great pain in the head and was sleepy. Attempts at swallowing caused a tendency to recurrence of "fits."

The patient got through the spring and summer fairly well. From time to time he had slight recurrence of the fits, generally induced by overexertion, or by some very trifling excitement such as that caused by his attending a religious service or his being visited by his friends. And, as before, they were followed by inability to speak or to move his legs, which passed off after a longer or shorter time; but in regaining power of locomotion he passed through a stage in which he did not appear to have full control over his legs, as they are described as "flying about very curiously." He complained, too, of a peculiar feeling at the base of the tongue, of a sense of constriction round the waist, and of shooting and dragging pains in the right leg, and of pins and needles in both hands. There was no anæsthesia, but he often said that his right side was cold, and any exertion caused the most intense pain on the top of his head, described as "sickening."

Early in November he was seen by some of the official visitors, and in consequence of an unguarded and uncomplimentary remark from one of them he was made very ill. He fainted instantly, and was with difficulty brought round. Later in the day this was followed by laughing fits with jactitation. The next day he could not move his legs or speak, although he quite knew what was said to him and what he wanted to say. In five weeks' time he was able to speak, but this return of articulation was transient, and vanished again without any renewed seizure, sometimes being regained at intervals. It was not until two months had elapsed from the shock that he could talk fairly, and during all this time he had been laid by in consequence of loss of power in the right leg.

During the next year, 1894, the patient's health certainly improved. The fits were few and far between, not so severe, and their sequelæ slighter and more transient. As a rule he could walk about pretty briskly and talk easily. But no week passed without the occurrence of some difficulty of speech, sometimes induced by a little overexertion of body or mind, or excitement, or sometimes as far as could be discovered occurring causelessly. The difficulty in speech was aphonia pure and simple. In November he had an interesting seizure after an evening party. It began with some loss of power in his legs: he walked in a queer, jerky way, with them crossed, and staggered greatly; his pupils were widely dilated, and he was quite conscious for a time and fought against the attack. Then unconsciousness came on, with a cry very like the epileptic cry, and he had to be carried to bed. About this time his discs were examined by Mr. Cross, and no trustworthy indications of past or present organic mischief were found. The patellar tendon reflex was now generally normal on both sides, and the action of heart and lungs was quiet and steady.

Throughout 1895 he was on the whole much better. The least excitement or over-exertion would induce an attack of hysterical laughter, followed by paresis and aphonia; but the attacks certainly became slighter and more transitory, and clonic convulsion or jactitation, the epileptic element, was absent. In fact, the course of his symptoms during the last two or three years has followed the course epitomised by a typical fit of hystero-epilepsy; viz., the predominance at first of symptoms mainly epileptic, or at all events of epilepsy associated with hysteria, but giving place at the close to symptoms ascribable to hysteria pure and simple, a sequence of events that supports the view that hystero-epilepsy is not a distinct pathological entity.

At the present time he is pretty comfortable, though often suffering from pain somewhere about the head and at the umbilicus, which latter may be quite independent of constipation. Hysterical seizures, followed by more or less paralysis in legs and larynx, are still readily induced, though more transient. He frequently suffers from umbilical pain and headache, whilst his bowels are sluggish and irregular, and he cannot sleep without a hypnotic. His pupils are sometimes unequal.

Before giving my opinion of the case, it may be convenient to briefly summarise the foregoing account of the symptoms.

The patient, a young man about 30, the son of a father almost certainly epileptic. Previous history good, save a vague story of some kind of fit when he was a lad.

Prominent symptoms: Tremors and convulsions, followed by defect of speech and some paresis. The tremors, &c., were of three kinds, viz.:

1. A tremor, to some degree rhythmical, affecting the muscular system generally and the eyeballs, though not involving the muscles of the head. This was increased by exertion and on observation, but shortly disappeared and did not reappear.

2. Violent jactitations, nearly always following headache, associated with loud laughter and other hysterical symptoms.

3. Tonic and clonic convulsions, producing a seizure indistinguishable from that of epilepsy (unless reliance is placed upon the fact that there was as a rule no cry, that the tongue was not bitten, or evacuations passed). Unconsciousness was complete, and much sleepiness or stupor succeeded.

Both 2 and 3 were followed by much exhaustion, and by loss of speech and of power of locomotion, lasting from a few hours to some months.

Other symptoms not to be lost sight of were: (1) increased patellar tendon reflex, on both sides at first; (2) dyspnœa and cardiac disturbance; (2) anomalous sensations at the base of the tongue; (4) shooting pains in the right eye; (5) cutting and shooting pains in the right thigh and leg; (6) a sense of constriction round the waist, and frequent umbilical pain generally concurring with pain at the vertex.

The important question to decide was, whether the patient was suffering from some organic disease of the cerebro-spinal system or not. He had certainly been condemned to death by many doctors prior to admission, and the early symptoms shortly after supported their view. The tremors and nystagmus and well-marked paresis strongly suggested disseminated sclerosis, while the jerkings of the limbs might have been referable to congestion or inflammation of the membranes of the cord. We find, too, in this disorder the increased tendency to laugh and cry causelessly that was conspicuous in our patient, while the convulsions, often unilateral and accompanied by hemiplegia, closely simulated the epileptiform attacks seen in disseminated sclerosis. For some time, I confess, I so regarded the case. But as time went on, and as many of these symptoms grew small by degrees and beautifully less until their urgency disappeared, another aspect of the case forced itself upon me. The hysterical element had of course always been noticeable; and noticeable, too, was the fact that the fits, whether of laughter with jactitations, or of epileptic convulsions, were certainly often caused by a mental or emotional disturbance or agitation, following, as they did, perhaps an unfeeling remark, perhaps a religious service, perhaps the visit of relatives: the *exciting cause was more often psychical than not.* At the present time the patient is free from every symptom of organic cerebro-spinal disease, unless significance is placed upon the slight inequality of pupils. And after having had the opportunity of watching the course of the disease for nearly three and a-half years, and of marking the disappearance of most of the symptoms, the retention of nutrition and of muscular tone in limbs apparently long paralysed, and the improvement in general health and condition, I have no doubt I was mistaken. I believe the case to be an example of that combination of hysteria with epilepsy to which the convenient name of hystero-epilepsy has been given. As Bristowe says, the two affections, hysteria and epilepsy, appear to run into one another, and hystero-epilepsy forms the link between them. This explanation, it seems to me, most satisfactorily accounts for the phenomena I have described, as well as for the course of the disease. Although the jactitations may not have been of the amplitude so graphically delineated and illustrated in some text-books on nervous disorders, they were sufficiently striking and characteristic. The convulsions, as I have said, were indistinguishable from those of epilepsy, though, as is generally the case in hystero-epilepsy, the tongue was not bitten or evacuations passed. In this patient jactitations did not, as is usual, follow after clonic convulsions, but they replaced one another. On this hypothesis the paresis and aphonia must be regarded as hysterical, and their character was quite consistent with that view, while the vomiting, occasional passage of large quantities of urine, headache, and anomalous sensations and actual pains in throat, abdomen, and limbs, can fairly be referred to the same cause.

